# Non-Alcoholic Fatty Liver Disease Is Associated with Kidney Glomerular Hyperfiltration in Adults with Metabolic Syndrome

**DOI:** 10.3390/jcm10081717

**Published:** 2021-04-16

**Authors:** Manuela Abbate, Catalina M. Mascaró, Sofía Montemayor, Miguel Casares, Cristina Gómez, Lucia Ugarriza, Silvia Tejada, Itziar Abete, Maria Angeles Zulet, Antoni Sureda, J. Alfredo Martínez, Josep A. Tur

**Affiliations:** 1 Research Group in Community Nutrition and Oxidative Stress, University of the Balearic Islands-IUNICS, 07122 Palma de Mallorca, Spain; manuela.abbate@uib.es (M.A.); c.mascaro@uib.es (C.M.M.); sofiamf16@gmail.com (S.M.); luciaugarriza@gmail.com (L.U.); silvia.tejada@uib.es (S.T.); antoni.sureda@uib.es (A.S.); 2 Health Research Institute of Balearic Islands (IdISBa), 07120 Palma de Mallorca, Spain; 3 Radiodiagnosis Service, Red Asistencial Juaneda, 07011 Palma de Mallorca, Spain; casaresmiguel@gmail.com; 4 Clinical Analysis Service, University Hospital Son Espases, 07120 Palma de Mallorca, Spain; cristina.gomez@ssib.es; 5 Camp Redó Primary Health Care Center, IBSalut, 07010 Palma de Mallorca, Spain; 6 CIBER Physiopathology of Obesity and Nutrition (CIBEROBN), Institute of Health Carlos III (ISCIII), 28029 Madrid, Spain; iabetego@unav.es (I.A.); mazulet@unav.es (M.A.Z.); jalfredo.martinez@imdea.org (J.A.M.); 7 Department of Nutrition, Food Sciences and Physiology, Center for Nutrition Research, University of Navarra, IDISNA, 31008 Pamplona, Spain; 8 Cardiometabolics Precision Nutrition Program, IMDEA Food, CEI UAM-CSIC, 28049 Madrid, Spain

**Keywords:** eGFR, glomerular hyperfiltration, kidney, non-alcoholic fatty liver disease, NAFLD, metabolic syndrome

## Abstract

Background: Non-alcoholic fatty liver disease (NAFLD) is a risk factor for the development of chronic kidney disease (CKD), which is early marked by kidney glomerular hyperfiltration. However, the association of NAFLD with kidney glomerular hyperfiltration has not been tested so far in adults with metabolic syndrome (MetS). Aims: To assess the relationship between NAFLD and kidney glomerular hyperfiltration in adults with MetS. Methods: The study included 154 participants aged 40–60 years with MetS and NAFLD diagnosed by ultrasound. NAFLD was confirmed by MRI in 109 subjects. Participants underwent anthropometric measurements, and biochemistry testing. Estimated GFR (eGFR) was calculated using the CKD-Epidemiology Collaboration (CKD-EPI) formula; hyperfiltration was defined as eGFR ≥ 120 mL/min. Results: Participants with MRI-proven NAFLD showed a worse metabolic profile and higher levels of eGFR than those with no NAFLD. Presence of NAFLD and increased weight were independently associated with an increased probability of presenting hyperfiltration. Conclusions: The present study shows an association between kidney glomerular hyperfiltration and NAFLD in adults with MetS. Establishing an association between NAFLD and kidney glomerular hyperfiltration would help to earlier identify those patients at increased risk of CKD, who would benefit from an early intervention.

## 1. Introduction

Non-alcoholic fatty liver disease (NAFLD) is defined by the presence of excessive hepatic fat accumulation in patients with no previous history of alcohol abuse [1]. From a stage of bland steatosis, NAFLD can progress to non-alcoholic steatohepatitis (NASH), cirrhosis, end-stage liver disease, and hepatocarcinoma [2]. Patients with NAFLD have a significantly higher risk of developing type 2 diabetes [3], chronic kidney disease (CKD) [4], and cardiovascular disease (CVD) [5] as compared to the general population matched for age and gender, and might die from CVD or non-liver cancer before suffering a liver-related death [6,7].

CKD is defined by a reduced glomerular filtration rate (GFR) (<60 mL/min/1.73 m^2^) and/or markers of kidney damage, indicated by moderately increased albuminuria 9albumin-to-creatinine-ratio (UACR) 30–300 mg/g), severely increased albuminuria (UACR > 300 mg/g), or nephrotic-range proteinuria (UACR > 2200 mg/g) [8]. CKD can progress to loss of kidney function and ultimately result in kidney failure [9]. Importantly, before the initiation of CKD, there might be a phase of glomerular hyperfiltration, significantly associated with hyperinsulinemia, insulin resistance, and obesity [10,11], which might enhance albumin ultrafiltration and excretion [12], and eventually shift to progressive renal function loss, and ultimately kidney failure [13,14]. Patients with CKD, at any stage of the disease, are strongly predisposed to developing CVD and are more likely to die from cardiovascular events than to progress to kidney failure [15].

NAFLD and CKD share multiple cardiometabolic risk factors such as obesity, insulin resistance, impaired glucose tolerance, dyslipidemia, and hypertension [16]. In the last decade, studies aiming at exploring the association between NAFLD and CKD have produced consistent evidence on the increased prevalence of CKD in patients with NAFLD [4]. The association between NAFLD and CKD seems to be independent of common risk factors, and NAFLD may precede early loss of kidney function, which worsens as NAFLD progresses to later stages [17,18,19].

Glomerular hyperfiltration is strongly related to the same cardiometabolic risk factors which are common in both NAFLD and CKD [20]; it is often defined as the first stage of renal impairment [14], and could be used as an early screening tool for patients with NAFLD at risk of CKD. However, the association between glomerular hyperfiltration and NAFLD has been explored only in children [21].

The aim of the present cross-sectional study was to assess the association between NAFLD and glomerular hyperfiltration in adults with metabolic syndrome (MetS).

## 2. Methods

The present analysis used baseline data belonging to an ongoing, multicenter, prospective, randomized, parallel-group, intervention trial conducted in Spain, which assesses the role of customized dietary and physical activity intervention on the pathophysiological mechanisms that may affect changes in liver fat deposits and progression of NAFLD in patients with MetS.

## 3. Subjects

**Figure 1:**
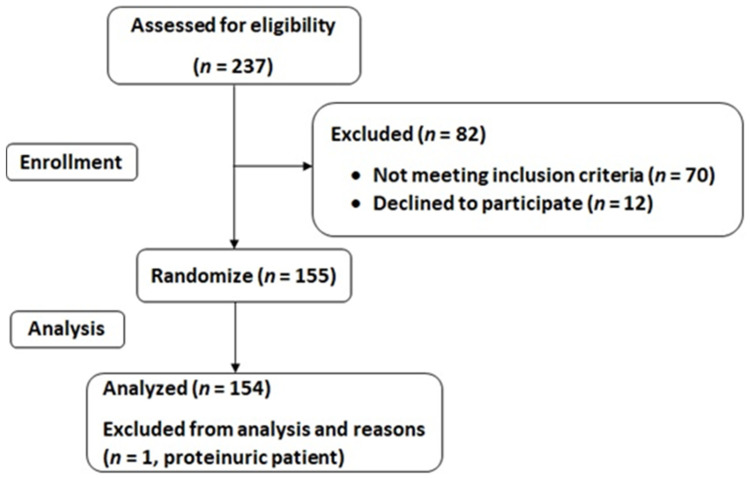
Study flow-chart.

The study included 155 participants aged between 40 and 60 years, with a diagnosis of NAFLD by means of ultrasound, with a body mass index (BMI) between 27 and 40 kg/m^2^, and meeting at least three of the five criteria of the MetS, as described in the International Diabetes Federation (IDF) consensus [22]. Participants were excluded if presenting the following exclusion criteria: previous cardiovascular disease; congestive heart failure; liver diseases (other than NAFLD); cancer or a history of malignancy in the previous five years; previous bariatric surgery; acute febrile illnesses; urinary tract infections, post-renal hematuria; hereditary or acquired hemochromatosis; severe albuminuria or nephrotic-range proteinuria; non-medicated depression or anxiety; alcohol and drug abuse and/or diagnosed alcohol use disorder; pregnancy; obesity associated with endocrinological diseases (other than medicated hypothyroidism); concomitant therapy with steroids; intense physical exercise; or unable to provide informed consent. At inclusion, all participants had a stable bodyweight during the previous six months. Figure 1 illustrates screening and selection criteria for study inclusion.

## 4. Ethics

The study protocol followed the Declaration of Helsinki ethical standards and all the procedures were approved by the Ethics Committee of the Balearic Islands (ref. IB 2251/14 PI) and by the Ethics Committee of the University of Navarra (ref. 054/2015mod2). All participants were informed of the purpose and the implications of the study and all provided the written consent to participate. The trial was registered at ClinicalTrials.gov with registry number NCT04442620 (https://clinicaltrials.gov/ct2/show/NCT04442620, accessed on 22 June 2020).

### 4.1. Anthropometric Measurements

Trained dietitians measured height to the nearest millimeter, with the participant’s head maintained in the Frankfurt Horizontal Plane, using a mobile stadiometer (Seca 213, SECA Deutschland, Hamburg, Germany). Weight and body fat were measured, with participants wearing light clothes and no shoes (0.6 kg of weight was subtracted for their clothing), using a Segmental Body Composition Analyzer for impedance testing (Tanita MC780P-MA, Tanita, Tokyo, Japan). BMI was calculated following the standard formula weight in kilograms divided by the square of height in meters. Three circumferences i.e., waist (WC), hip (HC), and neck (NC) were measured in duplicate with an anthropometric tape with the subjects standing upright. The average value of each measurement was used in the analysis. WC was measured halfway between the last rib and the iliac crest, HC was measured around the largest part of the hips, and NC was measured between the mid-cervical spine and the mid anterior neck. Blood pressure (BP) was measured in triplicate, in the non-dominant arm with a validated semi-automatic oscillometer (Omron HEM-705CP, Hoofddorp, The Netherlands) after 5 min of rest in a seated position. The average of three measurements, 2 min apart, was recorded.

### 4.2. General Data

During an initial interview with the study dietitian and study nurse, information on socioeconomic, medical history, current use of medication, and smoking status was collected. Information on alcohol consumption was collected by asking participants how many alcoholic beverages they consumed in a week on average, and responding either none, <7/week, or ≥7/week. Patients consuming ≥7 alcoholic beverages on average a week were excluded if presenting a drinking problem and/or a diagnosis of alcohol use disorder [23]. Physical activity habits over the previous 12 months were obtained using the validated Minnesota Leisure Time Physical Activity Questionnaire (Spanish version), which estimates total weekly energy expenditure in leisure time physical activity as metabolic equivalents of tasks (METs) using the following formula: METs·min·wk [24,25].

### 4.3. Adherence to the Mediterranean Diet

At the baseline interview, dietitians administered a 17-item Mediterranean Diet (MedDiet) questionnaire used in the PREDIMED trial [26], which assesses adherence to the MedDiet. Each item related to a specific dietary habit contemplated by the MedDiet and could be scored as 1 (compliance) or 0 (non-compliance). The total score ranged between 0 and 17 such as a score of 0 indicated no compliance and a score of 17 indicated maximum adherence.

### 4.4. Blood Collection and Analysis

Venous blood and single spot urine samples were collected in the morning after a 12-h overnight fast. Blood was collected through a venous catheter from the antecubital vein in suitable vacutainers containing ethylenediamine tetra-acetic acid (EDTA), citrate, or serum before immediate centrifugation at 3000 rpm for 10 min. Routine laboratory parameters such as fasting glucose, aspartate aminotransferase (AST), alanine aminotransferase (ALT), gamma-glutamyl transferase (GGT), uric acid, urea, creatinine, albumin, total cholesterol, high-density lipoprotein cholesterol (HDL-C), and triglycerides (TG), were measured in serum on the Abbott ARCHITECT c16000 (Abbott Laboratories, Abbott Park, IL, USA) employing specific commercial kits. Low-density lipoprotein cholesterol (LDL-C) was calculated according to the Friedewald formula [27]. Serum fasting insulin was assayed on the Cobas e411 platform (Roche, Switzerland), using either an enzyme-based electrochemiluminescence assay or an enzyme-linked immunosorbent assay kit. Serum ferritin was determined on the Chemiluminescent Microparticle Immunoassay (CMIA) automated analyzer ARCHITECT i2000 (CMIA, Abbott Core laboratory Systems, Lake Forest, IL, USA). Hematological parameters were analyzed in whole blood in an automatic flow cytometer analyzer (Cell-Dyn Sapphire platform, Abbott Core laboratory Systems, Lake Forest, IL, USA).

Urinary albumin excretion was measured from an early-morning urine sample as UACR. The urinary albumin concentration was determined by immunoturbidimetric assay and urinary creatinine concentration was measured by a modified Jaffe method on an Abbott ARCHITECT c16000.

Insulin resistance was estimated using the Homeostatic Model Assessment for Insulin Resistance (HOMA-IR) formula by Matthews et al. [28], as well as the TGs and glucose (TyG) index, calculated as the natural logarithm of the product of fasting plasma glucose and TG [29].

### 4.5. Estimated GFR

Estimated GFR (eGFR) was calculated using the new Chronic Kidney Disease Epidemiology Collaboration (CKD-EPI) equation, developed in 2009 [30]. The estimated renal function using the CKD-EPI equation is normalized for body surface area (BSA) and expressed as GFR mL/min/1.73 m^2^. Although the equation has been validated in populations with normal as well as low GFR, and is generally well-accepted [31], it has been argued that indexing eGFR for BSA in patients with increased weight can result in an underestimation of GFR, and masking a genuine association between renal function and body fat, such that it has been suggested that absolute estimates of GFR should be used instead [32,33,34]. Accordingly, eGFR was converted to absolute values (mL/min) by using the following formula [32]: (eGFR mL/min/1.73 m^2^ * BSA)/1.73 m^2^. BSA was calculated using the DuBois and DuBois equation [35]. Glomerular hyperfiltration was defined as Egfr ≥ 120 mL/min [36].

### 4.6. Imaging

Presence of hepatic steatosis was further assessed by abdominal MRI (Signa Explorer 1.5T, General Electric Healthcare, Chicago, IL, USA) by experienced radiologists. The protocol for the assessment of liver fat included the iterative decomposition of water and fat with echo asymmetry and least-squares estimation quantitation (IDEAL IQ) sequence. The chemical shift at particular echo times can be observed in gradient-echo imaging. The detection of fatty liver results from the degree of signal loss, which is proportional to the degree of lipid accumulation [37]. NAFLD was staged as absent (<6.4%) or present (≥6.4%), according to Tang et al. [38]. The gold standard for the diagnosis of NAFLD is by liver biopsy, nevertheless, MRI has been found to be a sensitive, highly accurate, and reliable non-invasive alternative [38].

## 5. Statistical Analyses

Statistical analysis was performed using SPSS statistical software package, version 25.0 (SPSS Inc., Chicago, IL, USA). Continuous variables are presented as mean ± SD while categorical variables are shown as counts (percentages). One patient who presented nephrotic-range proteinuria was excluded from the analysis to avoid biased parameter estimates and consequently biased test statistics and *p*-values.

Normality distributions for continuous data were determined using the Shapiro-Wilk test and visual inspection of histograms and normal probability plots. UACR, serum ferritin, fasting glucose, and TG presented a skewed distribution and were log-transformed before analysis; however, in the tables, they are presented as untransformed data for ease of interpretation.

One-way analysis of variance (ANOVA) or unequal variance *t*-test in the case of heterogeneity, for continuous variables, and χ^2^ test for categorical variables, were used to compare differences between participants with and without NAFLD measured by MRI. Post-hoc analysis for the χ^2^ tests was performed using the Bonferroni test.

Finally, multivariate logistic regression analyses were performed to assess odds ratios (ORs) and corresponding 95% confidence intervals (CI) of presenting hyperfiltration, while adjusting for potential confounders that showed a significant association in univariate analysis.

All *p*-values were two-sided, with *p* < 0.05.

## 6. Results

Characteristics of the study sample are shown in Table 1. The current analysis includes 154 patients with MetS, of which, 61 (39.6%) were women, 25 (16.1%) were current smokers, and 26 (16.9%) consumed ≥7 alcoholic drinks per week without presenting a drinking problem or a diagnosis of alcohol use disorder. The mean ± SD age was 52.3 ± 7.5. Light, moderate, and heavy physical activity was regularly done by 53 (34.6%), 25 (16.3%), and 9 (5.9%) patients, respectively. Of the 154 patients, 33 (21.4%) presented a diagnosis of T2DM at enrolment, while 62 (40.3%) suffered from high BP, and 47 (30.7%) were taking angiotensin converting enzyme (ACE) inhibitors or angiotensin II receptor blockers (ARBs) as an antihypertensive therapy.

All the 154 participants showed a diagnosis of NAFLD by ultrasound at baseline. MRI could be performed on 149 patients, and NAFLD was confirmed in 109 (73.2%). Of those with MRI-confirmed NAFLD, 41 (37.6%) were women and 68 (62.4%) were men; the mean ± SD age was 51.69 ± 7.36.

Clinical and laboratory characteristics of the study population divided by NAFLD status according to the MRI test are compared and displayed in Table 2. Mean liver fat % was 4.32 ± 1.50 in patients without NAFLD, and 17.05 ± 10.97 in patients with NAFLD. As compared with patients without NAFLD, those with NAFLD had generally higher WC, HC, weight, fat mass (%), fasting glucose, fasting insulin, HbA1c, HOMA-IR, TyG ratio, TG, and lower levels of HDL-c (*p* < 0.05). Compared to patients without NAFLD, those with NAFLD also had higher levels of ALT and AST, uric acid, and serum ferritin (*p* < 0.05). Finally, eGFR (adjusted and unadjusted for BSA) was higher in patients with NAFLD compared to patients without (*p* < 0.05), and the distribution of patients having an eGFR ≥ 120 mL/min was significantly higher in the NAFLD group (*n* = 42, 73.2%) when compared to any other group (*p* = 0.03). Levels of UACR presented increasing trends across the two groups, however, it did not reach statistical significance (*p* = 0.35). There were no significant differences between the two groups for age, BMI, systolic and diastolic BP, HR, total cholesterol, LDL-C, GGT, physical activity levels (expressed as METs), and adhesion to the MedDiet.

The percentage of patients with T2DM was higher in the group with NAFLD (*n* = 28, 87.5%) than in the group without NAFLD (*n* = 4, 12.5%) (*p* = 0.04); no differences were found in the prevalence of high BP, use of ACE inhibitors or ARBs, smoking habits, and alcohol consumption between the two groups. Twenty-six (16.9%) patients consumed ≥7 alcoholic drinks per week without presenting a drinking problem or a diagnosis of alcohol use disorder (diagnosed by their primary health care physicians).

Table 3 shows crude and adjusted OR (95% CI), respectively, univariate and adjusted logistic analysis, of presenting hyperfiltration. Univariate analysis showed that increased odds of having an eGFR ≥ 120 mL/min was associated with the presence of NAFLD (OR 5.126; 95% CI 1.473–17.831), male gender (OR 4.583; 95% CI 1.779–11.810), and weight (OR 1.088; 95% CI 1.050–1.128), whilst female gender (OR 0.218; 95% CI 0.085–0.562) and age (OR 0.946; 95% CI 0.898–0.996) were associated with a reduced probability of presenting hyperfiltration. When variables were entered simultaneously in the logistic regression model, the presence of NAFLD (OR 3.901; 95% CI 1.043–14.502) and weight (OR 1.068; 95% CI 1.027–1.111) remained significantly associated with increased odds of hyperfiltration. Not presenting NAFLD, on the other hand, was associated with a reduced probability of having hyperfiltration (OR 0.256; 95% CI 0.069–0.953).

## 7. Discussion

The present cross-sectional analysis showed that MetS adult patients with NAFLD showed a worse cardiometabolic profile and higher levels of eGFR compared to patients without NAFLD. Moreover, NAFLD and weight were associated too with increased odds of hyperfiltration.

Numerous studies have shown a direct association between increased risk of incident CKD and NAFLD. The reported prevalence of CKD in NAFLD ranges between 20–50%, compared with 5–30% in patients without NAFLD [4]. In a recent meta-analysis including longitudinal and cross-sectional studies for a total of 29,282 patients, NAFLD was associated with an increased risk of prevalent and incident CKD (defined as eGFR < 60 mL/min/1.73 m^2^ and/or nephrotic-range proteinuria). In turn, CKD severity was associated with the progression of NAFLD to more advanced stages [17], and this association was independent of T2DM. In a community-based cohort study including 8329 non-diabetic, non-hypertensive working men, with normal kidney function at baseline, 324 of them developed incident CKD during a follow-up of 3.2 years. NAFLD was independently associated with an increased risk of incident CKD [18]. According to experimental evidence, NAFLD may worsen systemic and hepatic insulin resistance, lipid metabolism, inflammation, and oxidative stress, which play an important role in the development of CKD [16].

To the best of our knowledge, evidence of an association between NAFLD and glomerular hyperfiltration is available only in just a very recent study on a pediatric cohort. This study [21] observed that in 179 obese children with histologically confirmed NAFLD and aged 12–16 years, 20% had glomerular hyperfiltration (>136 mL/min/1.73 m^2^), and 15% had low GFR (<90 mL/min/1.73 m^2^). Compared with normal eGFR, hyperfiltration was independently associated with a greater NAFLD activity score, after adjustment for age, sex, ethnicity, obesity severity, presence of T2DM, as well as medications.

Hyperfiltration in adults has been extensively contemplated in MetS [11,20], as a risk factor for CKD; up to 73% of T2DM patients [39], and 40% of patients with MetS [11] with hyperfiltration, will eventually develop CKD. Hyperfiltration might enhance albumin ultrafiltration and excretion [12,40], and eventually shift to a phase of progressive loss of renal function, which, paralleled to a further rise in albuminuria, ultimately leads to kidney failure [13,14]. In T2DM, hyperfiltering subjects experienced an accelerated GFR decline compared to non-hyperfiltering [41]. The development of hyperfiltration is strongly influenced by increased glycemia, high blood pressure, and, generally, the MetS [10,42]. Moreover, in a recent meta-analysis, renal hyperfiltration was associated with an increased risk of cardiovascular disease and all-cause mortality in populations including healthy individuals as well as patients with T2DM [18]. Most importantly, weight loss, achieved either through caloric restriction or bariatric surgery can significantly reduce glomerular hyperfiltration and concomitant albuminuria, and restore normal renal function, as well as improving a series of cardiometabolic risk factors also associated with the onset and progression of CKD [43,44].

In the current study, patients with NAFLD presented higher levels of eGFR as well as a significantly increased prevalence of hyperfiltration (73.2%) compared to patients without NAFLD; patients with NAFLD also presented a worse metabolic profile, which, according to previous studies, can influence the development of hyperfiltration as well as CKD [10,16,20,43]. Moreover, NAFLD and increased weight were associated with an increased probability of presenting hyperfiltration.

Establishing an association between NAFLD and glomerular hyperfiltration would help to earlier identify those patients at increased risk of CKD, who would benefit from an early intervention. It has already been suggested that individuals with NAFLD should be screened for CKD by eGFR and/or urinary albumin excretion even when classical risk factors for CKD are absent [17]; nevertheless, as hyperfiltration often precedes CKD, screening for an abnormally elevated eGFR could allow for an even earlier recognition of a possible risk.

## 8. Strengths and Limitations

The strengths and limitations of this study were as follows: as for the former, to the best of our knowledge, this is the first evidence of an association between NAFLD and glomerular hyperfiltration in adults. Moreover, images were obtained by MRI which is considered the most sensitive and accurate non-invasive method for quantifying liver fat [45]. Limitations, on the other hand, include the design of the study and the limited number of patients. The study was not specifically designed to look at the predictive effect of NAFLD on eGFR, and participants were not selected according to stages of eGFR. A bigger sample of patients could give a more confident answer to the possible relationship between NAFLD and eGFR at different stages in a population with MetS.

## 9. Conclusions

Results from the current study show that NAFLD and weight are associated with glomerular hyperfiltration in patients with MetS. Screening patients with NAFLD for kidney glomerular hyperfiltration would help to earlier identify those patients at increased risk of CKD, who would benefit from early intervention.

## Figures and Tables

**Table 1:** Characteristics of the study sample.

Variables	*n* (%)
*n*	154
Female	61 (39.6)
Of which have the menopause	33 (54.1)
Age (y) (mean ± SD)	52.27 ± 7.46
Marital Status	
Single	14 (9.1)
Married/domestic partnership	116 (75.3)
Divorced/separated/widowed	24 (15.6)
Employment	
Working	115 (74.7)
Unemployed/retired/housewife	39 (25.3)
Education Level	
University/post-university	44 (28.6)
Secondary education	61 (39.6)
Primary education	41 (26.6)
None	8 (5.2)
Currently smoking	25 (16.1)
Alcohol ≥ 7 drinks/w	26 (16.9)
Regular Physical Activity	
None	66 (43.1)
Light	53 (34.6)
Moderate	25 (16.3)
Heavy	9 (5.9)
T2DM	33 (21.4)
High BP	62 (40.3)
Concomitant drugs	
Hypoglycemic agents	
Any	30 (19.6)
Oral hypoglycemic agents alone	26 (16.9)
Insulin and oral hypoglycemic agents	4 (2.6)
Antihypertensive Agents	
Any	53 (34.4)
Diuretic	17 (11.1)
β-Blocker	8 (5.2)
Calcium-channel blockers	9 (5.9)
ACE inhibitors/ARBs	47 (30.7)
Lipid-Lowering Agents	
Any	43 (28.1)
Statin alone	27 (17.6)
Fibrate alone	8 (5.2)
Statin and fibrate	4 (2.6)
Antiplatelet agent	7 (4.6)
Concomitant Drugs–Other	
Any	82 (53.6)
Thyroid medications	10 (6.5)
Depression/anxiety/insomnia	26 (17.0)
Gout medications	10 (6.5)

Data are expressed as count (%), unless otherwise specified. SD = standard deviation; T2DM = type 2 diabetes mellitus; BP = blood pressure; ACE = angiotensin converting enzyme; ARBs = angiotensin II receptor blockers.

**Table 2:** Clinical and laboratory characteristics of the study population according to presence/absence of NAFLD.

	NO NAFLD	NAFLD	*p*
*n*	40	109	
Age	53.2 ± 7.4	51.7 ± 7.4	0.280
**Anthropometric Variables**			
Waist circumference (cm)	91.6 ± 11.4	96.5 ± 13.4	0.040
Hip circumference (cm)	107.2 ± 8.4	113.6 ± 8.7	<0.001
Weight (kg)	32.1 ± 3.2	34.2 ± 3.7	0.002
BMI (kg/m^2^)	34.9 ± 7.1	35.7 ± 6.9	0.530
Fat mass (%)	91.6 ± 11.4	96.5 ± 13.4	0.040
**Clinical Parameters**			
Systolic BP (mmHg)	131.1 ± 14.8	136.5 ± 15.0	0.050
Diastolic BP (mmHg)	85.4 ± 8.1	85.2 ± 9.7	0.880
HR (bpm)	67.6 ± 10.4	71.6 ± 11.4	0.060
**Metabolic Variables**			
Fasting glucose (mg/dL)	104.3 ± 20.2	119.1 ± 47.0	0.007
Fasting insulin (μUI/mL)	14.5 ± 6.0	21.7 ± 10.7	<0.001
HbA1c (%)	5.7 ± 0.6	6.2 ± 1.4	0.001
HOMA-IR	3.7 ± 1.7	6.5 ± 4.0	<0.001
TyG	4.8 ± 0.2	5.0 ± 0.3	0.001
**Blood Lipids**			
Total cholesterol (mg/dL)	191.4 ± 38.6	202.1 ± 51.0	0.230
HDL-cholesterol (mg/dL)	47.6 ± 13.2	43.1 ± 10.1	0.030
LDL-cholesterol (mg/dL)	114.0 ± 32.2	116.8 ± 35.6	0.670
Triglycerides (mg/dL) ^#^	149.2 ± 70.2	221.4 ± 245.8	0.003
**Hepatic Variables**			
NAFLD (mean fat %)	4.3 ± 1.5	17.0 ± 11.0	<0.001
ALT (U/L)	22.6 ± 9.7	41.5 ± 33.6	0.001
AST (U/L)	20.1 ± 4.6	28.0 ± 14.5	0.001
GGT (U/L)	35.6 ± 26.2	54.1 ± 61.2	0.070
**Renal Variables**			
UACR (mg/g)	11.7 ± 13.3	17.7 ± 37.3	0.350
UACR < 30 mg/g (*n* (%))	32 (22.7)	90 (63.8)	0.990
UACR 30–300 mg/g (*n* (%))	5 (3.5)	14 (9.9)	
eGFR (mL/min/1.73 m^2^)	104.3 ± 20.1	116.9 ± 21.6	0.002
eGFR (mL/min)	121.5 ± 24.4	139.0 ± 32.7	0.002
eGFR < 90 mL/min (*n* (%))	17 (42.5) *	28 (25.7)	0.030
eGFR 90–120 mL/min (*n* (%))	15 (37.5)	39 (35.8)	
eGFR ≥ 120 mL/min (*n* (%))	8 (20.0)	42 (73.2) *	
**Other Variables**			
Uric acid (mg/dL)	5.6 ± 1.4	6.12 ± 1.5	0.070
Serum ferritin (ng/mL)	111.0 ± 100.3	167.16 ± 155.1	0.020
METs (min/week)	3406 ± 2864	3220 ± 3358	0.760
MedDiet adhesion	7 ± 3	7 ± 3	0.710

Data are expressed as mean ± standard deviation unless otherwise stated; BMI = body mass index; BP = blood pressure; HR = heart rate; MAP = mean arterial pressure; TyG = triglycerides and glucose index; NAFLD = non-alcoholic fatty liver disease; MPV = mean platelet volume; UACR = urinary albumin-to-creatinine ratio; eGFR = estimated glomerular filtration rate; METs = metabolic equivalents; MedDiet = Mediterranean diet; * = the percentage of patients in the two reported groups were significantly higher when compared to any other group according to the Bonferroni post-hoc test for χ^2^ test; ^#^ = log-transformed.

**Table 3:** Odds ratios of presenting hyperfiltration (eGFR > 120 mL/min) in patients with MetS.

Variables	OR Crude (95% CI)	*p*	OR Adjusted (95% CI)	*p*
Age	0.946 (0.898–0.996)	0.030	0.979 (0.923–1.038)	0.470
Gender		0.002		0.102
Male	4.583 (1.779–11.810)		2.375 (0.843–6.690)	
Female	0.218 (0.085–0.562)		0.421 (0.149–1.187)	
NAFLD		0.010		0.042
No	0.195 (0.056–0.697)		0.256 (0.069–0.953)	
Yes	5.126 (1.473–17.831)		3.901 (1.043–14.502)	
Weight (kg)	1.088 (1.050–1.128)	<0.001	1.068 (1.027–1.111)	0.001

OR = odds ratio; NAFLD = non-alcoholic fatty liver disease.

## Data Availability

There are restrictions on the availability of data for this trial, due to the signed consent agreements around data sharing, which only allow access to external researchers for studies following the project purposes. Requestors wishing to access the trial data used in this study can make a request to pep.tur@uib.es.
